# Imaging Software Programs for Reliable Mathematical Measurements in Orthodontics

**DOI:** 10.3390/dj8030081

**Published:** 2020-08-03

**Authors:** Eman Saad Radwan, Andrea Scribante, Maria Francesca Sfondrini, Mona A Montasser

**Affiliations:** 1 Orthodontic Department, Faculty of Dentistry, Mansoura University, Mansoura 35516, Egypt; Dr.EmanSaad@hotmail.com; 2 Unit of Orthodontics and Paediatric Dentistry–Section of Dentistry–Department of Clinical, Surgical, Diagnostic and Paediatric Sciences, University of Pavia, 27100 Pavia, Italy; francesca.sfondrini@unipv.it

**Keywords:** Orthodontics, software program, mathematical measurements

## Abstract

Aim: To evaluate the reliability of linear and angular measurements taken using different software programs in orthodontics. Materials and Methods: A sample of four software programs from different manufacturers, namely MicroDicom viewer, Photoshop^®^ CS3, AutoCAD^®^, and Image-Pro^®^, were used for measuring the geometric features of four types of miniscrews from different manufacturers. Each miniscrew type presented a group: Group I, Tomas^®^ (Dentaurum, Ispringen, Germany); Group II, HUBIT^®^ (HUBIT, Gyeonggi-do, Korea); Group III, AbsoAnchor^®^ (Dentos, Daegu, Korea); and Group IV, Creative (Creative, Zhejiang, China). Measurements of apical face angle, thread angle, lead angle, flank, pitch depth, and width were taken on 45 × magnification scanning electron microscope images of the shafts of the miniscrews. One assessor measured the seven geometric features for the four types of miniscrews using the four software programs twice in two sessions separated by a three week interval. Results: Pairwise comparisons, for each of the four miniscrew groups, showed that the only common result observed was the significant difference (*p* < 0.001) between measurements of flank taken by the four software programs. When measurements of the four types of miniscrews were pooled into one group, a high degree of intra-rater reliability (ICC range from 0.9 to 1.0) for all the seven geometric features was found with all the four software programs. The paired *t*-test showed insignificant difference (at *p* ≤ 0.05) between the first and second measurements, except for a few measurements including pitch width measured by Image-Pro^®^ (*p* = 0.012), MicroDicom (*p* = 0.023), and Photoshop^®^ (*p* = 0.001). Conclusions: Results did not give absolute superiority to one software program over the others and suggested an assessor effect. Assessor estimates could have been affected, among other factors, by the design of the miniscrews and the technical features of the software programs.

## 1. Introduction

Different instruments [1,2] and software programs [3] are available for mathematical measurements. Medical and dental software programs with a wide range of applications in clinical and research practices are now affordable and within reach of everyone [4,5,6]. Software programs for anatomical identifications and measurements largely replaced manual or anthropometric methods to save time and effort and sometimes because of allegedly increased precision, accuracy, and/or reliability, or wishfully, for both reasons [7,8]. Using software programs is sometimes inevitable when measurements of mini-and micro-structures are required [9]. Previous studies [10,11,12] compared manual versus digital measurements and evaluated the accuracy and reliability of software programs. This was done on both soft and hard structures. In a study that compared soft tissue measurements obtained using software programs for surgical purposes to direct clinical measurements, different software programs showed varied accuracy and reproducibility [10]. In a study that assessed the accuracy and reliability of imaging software for measurements of upper airway from cone beam computed tomography CBCT images, Chen et al. [11] compared three software programs and found different accuracies of the volume, length, and cross section. Another study about soft tissue measurements’ accuracy compared Dolphin imaging software^®^ and nasopharyngoscopy resulted in weak support for the use of the software for volume and minimal cross-sectional airway measurements. The software measurements from CBCT scans did not correlate well with the measurements from nasopharyngoscopy [12]. A research group [13] that designed a software program for automated identification of craniofacial landmarks on CBCT images measured the distances of coordinates for any of the landmarks to evaluate the software accuracy. Another group [14] that opted to design its software program evaluated measurements of subcutaneous adipose tissue using software for semi automated measurements of subcutaneous adipose tissue and found high accuracy and reliability of measurements obtained by well trained assessors.

Previous studies measured the dimensions of different components of orthodontic appliances including brackets [15], archwires [16], and miniscrews [17,18,19]. The purpose of these studies was to evaluate the accuracy of the measured dimensions in comparison to the dimensions given by the manufactures or to detect the correlation between dimensions and the mechanical performance of the studied orthodontic components. Linear and angular measurements of small size objects present additional difficulty because measurements on a microscopic scale are needed. For these purposes, scanning electron microscope (SEM) is widely used because it gives magnified images of high resolution, which makes micro measurements possible. Recognized as a valuable scientific instrument, multiple factors that could affect the measurements taken with the SEM have been studied; these factors include the acquisition of the image, the SEM calibration, and the effects of specimen contamination [20,21,22]. Because of the accuracy and reliability concerns, scientists studied these factors and tried to develop methods to improve the technique and overcome its shortcomings [9,23,24].

It is evident from the above studies that software programs have become increasingly used for mathematical measurements in the medical field. Digital measurements of soft and hard tissues and measurements of devices and components of appliances are all needed in orthodontics for diagnosis, treatment planning and evaluation, biomechanical choices, as well as in related research. The impetus behind the current study was the observed lack of evaluation of software packages as an influencing factor on the accuracy and/or reliability of mathematical measurements. Very few studies have evaluated the accuracy and reliability of different software packages for mathematical measurements [11]. Therefore, this study was conducted to evaluate and compare the reliability of linear and angular measurements taken by different software programs used in orthodontics.

## 2. Materials and Methods

Miniscrews: Four types of miniscrews from different manufacturers were used in this study. Each type presented a group: Group I, Tomas^®^ (Dentaurum, Ispringen, Germany); Group II, HUBIT^®^ miniscrew (HUBIT, Gyeonggi-do, Korea); Group III, AbsoAnchor^®^ (Dentos, Daegu, Korea); and Group IV, Creative (Creative, Zhejiang, China). Each group included 10 miniscrews. Although the miniscrews from the different manufacturers had different geometric linear and angular measurements, all had conical shafts of 1.6 mm diameter and 6.0 mm length.

**Figure 1:**
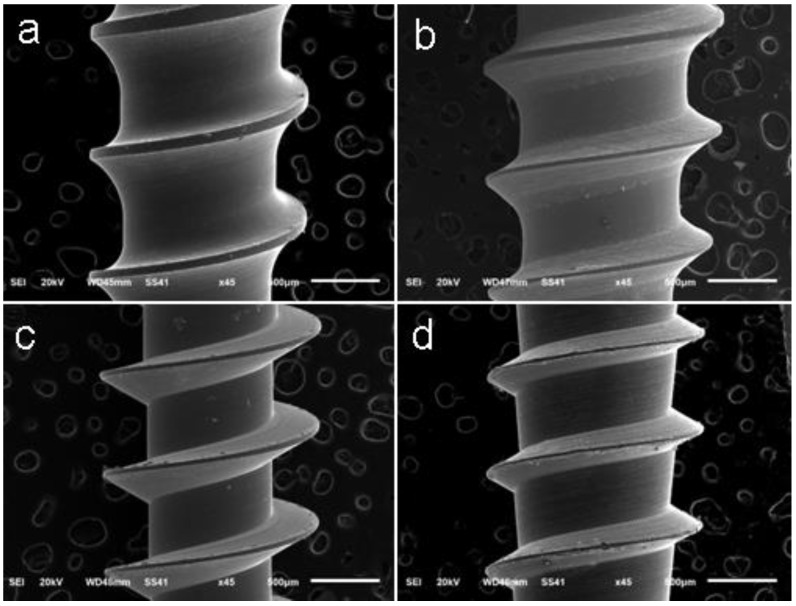
Scanning electron microscope (SEM) images showing the thread details of the miniscrews: (**a**) Tomas^®^, (**b**) HUBIT^®^, (**c**) AbsoAnchor^®^, and (**d**) Creative at 45 × magnification.

SEM images: The study used SEM (model JSM-6510LV; JEOL, Tokyo, Japan) for 3-dimensional imaging of the shafts of the miniscrews on a micrometric scale. The scans were done at 45 × magnification (Figure 1).

Software programs: Four software programs from different manufacturers were used in this study: MicroDicom viewer (MicroDicom, Sofia, Bulgaria), Photoshop^®^ CS3 (Adobe Inc., San Jose, CA, USA) AutoCAD^®^ 2010 (Autodesk, San Rafael, CA, USA), and Image-Pro^®^ (Media Cybernetics, Rockville, MD, USA).

**Figure 2:**
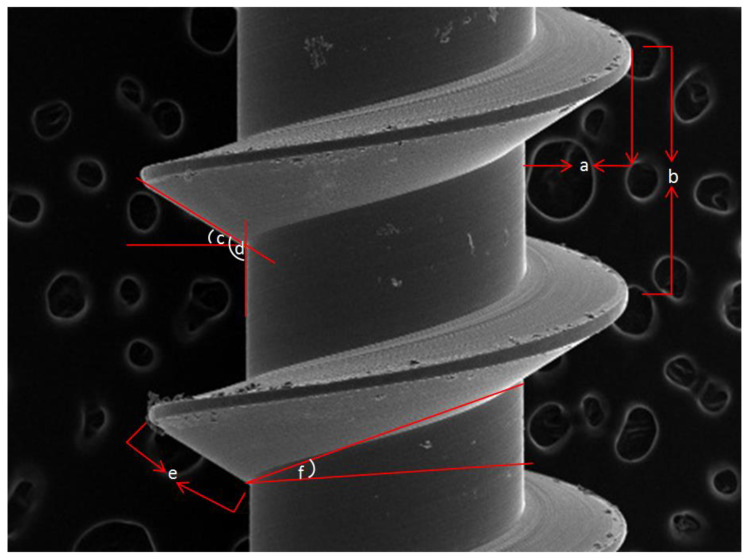
Linear and angular measurements of the geometric features of the miniscrews; (**a**) Thread depth: the distance from the tip to the root of the thread measured perpendicular to the longitudinal axis, (**b**) Thread pitch: the distance from the center of one thread crest to the center of the next. (**c**) Thread angle: the angle between the flank of a thread and the surface adjacent to it, (**d**) Apical face angle: the angle between the flank of a thread and perpendicular to the axis of a thread (**e**) Flank of the thread: the surface of thread that connect the tip with the root of the thread. (**f**) Lead angle: the angle made by the helix of the thread with a plane perpendicular to the axis. This is measured in an axial plane. Thread shape factor (TSF): calculated as the percentage between the mean thread depth and pitch.

Measurements: The same linear and angular measurements (Figure 2) were taken for each miniscrew in each of the four groups. Apical face angle, thread angle, and lead angle were measured. Linear measurements included: flank, pitch depth, and pitch width. Definitions and details of the measurements have been given in previous publications [19,25].

Measurements were done after calibration of the image in each program as the scale of image had to be adjusted. To calibrate an image, calibration marks were placed on two points that were a known distance apart and by entering the actual distance into the program. The assessor measured the seven geometric features of the four types of miniscrews using each of the four software programs. Measurements were done in two sessions separated by a three week interval.

Statistical analyses: Descriptive statistics included minimum, maximum, mean, and standard deviation. The Kolmogorov-Smirnov test verified the normality of the data distribution. F-test (ANOVA) was used for the normally distributed quantitative variables to compare between the groups, and Tukey’s Post-Hoc test was used for pairwise comparisons. Significance was set at the 5% level. Intraclass correlation coefficient (ICC) and paired *t*-test were used for reliability assessment. An ICC form that included a two-way random effects model, single assessor type, and consistency was selected to be applied for the results of the current study. Mean estimates and the 95% confidence intervals (CI) had been reported for each ICC. The ICCs had been interpreted using a system suggested by McGraw and Wong [26] as follows: less than 0.75 Z, poor agreements; 0.75 to less than 0.90 Z, moderate agreements; 0.90 or greater Z, high agreements. One of the well known and most commonly used normalization techniques is Fisher’s Z transformation [27]. *p* value less than 0.05 was considered statistically significant. Data were analyzed with SPSS software package version 20.0 (IBM Corp: Armonk, NY, USA).

## 3. Results

Table 1, Table 2, Table 3 and Table 4 show the minimum, maximum, mean, standard deviation, and CI of the selected miniscrews’ geometric feature measurements. Each Table shows the descriptive statistics of the measurements for one group of the miniscrews taken by the four included software programs. Each Table also shows the results of one-way ANOVA and Post Hoc Tukey’s test. The only common result observed was the significant difference (*p* < 0.001) of flank measurements between the different software programs in each of the four miniscrew types.

The ICC for intra-rater reliability ranged between poor and high, while the paired *t*-test showed, except for a few readings, an insignificant difference between the two rounds of measurements (*p* ≤ 0.05), Table 5, Table 6, Table 7 and Table 8. When the variables were combined for all the four types of miniscrews (Table 9), a high degree of intra-rater reliability for all the seven geometric features was found with all the four software programs; the value of ICC ranged from 0.9 to 1.0. The *t*-test showed significant difference between the first and second measurements of pitch width measured by Image-Pro^®^ (*p* = 0.012), MicroDicom (*p* = 0.023), and Photoshop^®^ (*p* = 0.001).

## 4. Discussion

Mathematical measurements in this study could have been affected by the scanned images, the software program, and/or the assessor. Frederick et al. [2] tested the accuracy of SEM linear measurements on dental implants and found values within a margin of the values given by the manufacturer. They suggested a similar performance of the SEM, Optical Microscope, and Micro-Computed Tomography. Accuracy of measurements taken by different software programs had been investigated before; many studies in the medical field have been carried out on soft tissue measurements. Quieregatto et al. [10] compared the precision and accuracy of measurements obtained with AutoCAD^®^, ImageTool^®^, and Photoshop^®^ with reference to clinical soft tissue measurements. Precision, which reflects reproducibility of measurements, was best with AutoCAD^®^ and lowest with ImageTool^®^, while Photoshop^®^ showed intermediate precision. Another study [11] on upper airway measurements using three software programs found high reliability of the three software programs. However, they reported inaccurate measurements; the three programs underestimated all tested parameters.

In the current study, when comparing the mean values of each geometric feature in each miniscrew group between the four software programs (Table 1, Table 2, Table 3 and Table 4), there was no specific pattern noticed. The group that included Tomas^®^ miniscrews showed significant differences between the four programs for all the features measured except pitch width. Curved and indefinite points and line angles characterized the Tomas^®^ miniscrews, therefore variations could have happened when locating the points for drawing the lines and planes required for linear and angular measurements. Miniscrews in the other groups showed more definite points and line angles, in varying degrees, and so the statistical results were different among the groups. In this context, flank was the only feature that was significantly different in all the four miniscrew types. The scans showed characteristic indefinite points at the tip of the thread as in group I and group II or at both the tip and root of the thread as in groups III and IV, which made it difficult to locate the points required for flank measurement in a precise, repeatable way. The small size of the flank, which ranged from 228.000μ–464.832µ, should also be considered in this regard. Manufacturing specifications seemed to be a strong factor influencing the measurements obtained by each software program. Based on these results, we could not give superiority to one software program over the others.

Reliability tests are important to build confidence in measurements and consequently in any steps taken further based on these measurements. ICC could test inter-assessor, intra-assessor, or measurement-remeasurement reliability [28]. The current study depended on one assessor, which measured the seven geometric features of the four types of miniscrews using the four software programs twice in two sessions separated by three weeks. Therefore, reliability reflected variations in measurements between assessments made by the same assessor in different sessions.

In the current study, ICC and paired *t*-test were done; the ICC showed the strength of association between the two rounds of measurements, while the paired *t*-test disclosed differences between the first and second measurements. Previously, the intra-assessor reliability of mathematical measurements calculated using different software packages had shown varied reliabilities [5]. Interpreting the ICC results is very critical since there are no standard values agreed on to indicate the different degrees of reliability [25]. Lower ICC values in the current study had not for sure indicated greater variability [25,29]. The observed negative results of ICC (Table 5, Table 6, Table 7 and Table 8) could be attributed to lower differences between miniscrews rather than differences between the assessor estimates in the different sessions. These results emphasized the effect of the design of the measured object on the assessor estimates of the location of points and planes for measurements. Assessor estimates are important since these software programs are not self-reporting. Lower ICC values could also be due to a small sample size. Taking advantage of the low differences in the measurements between the four groups of miniscrews, the measurements of each variable for the four miniscrews’ types were combined (Table 9) and the statistical analysis resulted in high intra-assessor reliability for the seven geometric features. Reliability was the key point of study in this research and because measurements could be reliable but not accurate, studies investigating the accurateness of the measurements are needed. These studies would need a gold standard for comparison; this gold standard would be the measurements from the manufacturers. The paired *t*-test showed insignificant differences (at *p* ≤ 0.05) between the first and second measurements, except for very few measurements.

The technical features of the software could affect assessor estimates and consequently the measurement’s reliability; technical features could positively influence the effectiveness and efficiency of the software or could do the opposite. Technical features differed between the four software programs; to give examples, Image-pro^®^ had a local zoom feature that was used to magnify a specific area to facilitate accurate position of points and drawing of lines when needed. Photoshop^®^ and AutoCAD^®^ enabled drawing perpendicular lines automatically, which was advantageous in contrast to MicroDicom where lines were drawn manually. Image-Pro^®^ displayed X and Y axes, which helped to draw perpendicular lines. The assessor estimates could therefore be affected by the design of the object, the technical features of the software program, and factors that may include, but are not limited to, computer familiarity and level of training on the software program. In the current study, MicroDicom individual measurements were the least precise as the software gave numbers rounded to a maximum of two digits, contrary to the other software programs that gave measurements rounded to more than five digits. However, it seemed that this feature did not influence the statistical comparisons. Nevertheless, this is a feature that might need to be considered if precision of measurements is important. Extreme precision is not always a plus; it may sometime complicate the reading or may give a misleading impression about the exactness of the measurements [30]. Scientific papers should above all be written in a comprehensive, logical way. Presenting data may sometimes become tricky due to needing to balance between accuracy and precision on one side and simplicity on the other hand, including rounding to a reasonable number of digits [31]. Precision and accuracy of measurements are two different parameters; precision indicates the information obtained from a number, while accuracy indicates the correctness of a number. In another way, precision indicates the reliability as seen in the repeatability of the measurements, while accuracy indicates the closeness of a measurement compared to the real measurement. Therefore, greater precision does not mean greater accuracy and the other way around. An instrument or device could produce the same inaccurate measurement over and over again. This concern is particularly important when planning miniscrew assisted palatal expanders or other miniscrew supported orthodontic devices with a completely digital workflow [32]. In these cases, the accuracy of the measurements is particularly important, thus allowing the clinician to choose diameter and type of miniscrew as these characteristics have a great influence on the mechanical behavior of the screws [33,34].

One of the biggest considerations when choosing a software package is its user friendliness. From our subjective experience with the four software packages, they differed greatly when judging their user friendliness quality. Some technical characteristics could influence the efficiency and effectiveness of the software and then its user friendliness—for example, it was possible to export measurements taken by Image pro to excel or text files. Also, Photoshop^®^ exported the outcome to a text file. However, MicroDicom only dealt with image files. Other general characteristics that make a software package attractive to users include installation and updating easiness, intuitiveness and simplicity of navigation, and technical support. User friendliness might be a key element when choosing software packages.

However, it was not one of our intentions to compare the user friendliness of the software packages in the current study.

As the mathematical measurements could be affected by the software program used, clinicians need evidence for the reliability of software programs for such applications, which would help them understand and compare software packages. Therefore, the focus in the present study was on the software programs. In the current study, miniscrews as one of the orthodontic appliance components was used, but applications of software programs for mathematical measurements in orthodontics also extend to hard and soft tissue measurements.

## 5. Conclusions

1. The results did not give absolute superiority to one software program over the others and reliability tests suggested an assessor effect.
2. Assessor estimates could have been affected by the design of the miniscrews, the technical features of the software programs, and other general factors.
3. Even when a software program did not give highly precise individual measurements, the reliability of the measurements was not compromised.
4. Studies focusing on the accurateness of the measurements are recommended.
5. Studies on soft tissues measurements are also needed.

## Figures and Tables

**Table 1:** Comparison between the measurements of (Group I) miniscrews calculated by the four software programs.

Group I (Tomas^®^)		AutoCAD^®^ (*n* = 10)	Image-Pro^®^ (*n* = 10)	MicroDicom (*n* = 10)	Photoshop^®^ (*n* = 10)	F	*p*
**Pitch depth (µm)**	(Min.–Max.)	(207.301–233.313)	(211.400–266.970)	(220.000–270.000)	(214.120–236.960)	3.766 *	0.019 *
	Mean ± SD	218.660 ^b^ ± 9.560	231.959 ^a,b^ ± 16.353	238.000 ^a^ ± 16.865	227.024 ^a,b^ ± 8.174		
**Pitch Width (µm)**	(Min.–Max.)	(860.538–958.160)	(871.600–953.460)	(870.000–890.000)	(866.630–960.800)	0.996	0.406
	Mean ± SD	894.116 ± 37.531	899.881 ± 32.121	881.000 ± 7.379	902.095 ± 33.227		
**TSF (%)**	(Min.–Max.)	(222.291–267.777)	(228.671–300.025)	(250.000–306.818)	(229.544–268.422)	3.713 *	0.020 *
	Mean ± SD	245.035 ^b^ ± 16.449	258.137 ^a,b^ ± 21.442	270.097 ^a^ ± 18.196	251.951 ^a,b^ ± 12.568		
**Flank (µm)**	(Min.–Max.)	(451.345–467.378)	(461.080–467.590)	(450.000–460.000)	(460.250–469.410)	20.985 *	<0.001 *
	Mean ± SD	462.126 ^a^ ± 4.676	464.150 ^a^ ± 1.949	453.000 ^b^ ± 4.830	464.832 ^a^ ± 2.847		
**Thread Angle (°)**	(Min.–Max.)	(143.022662–143.987038)	(146.012785–146.956282)	(146.090000–146.900000)	(146.016464–146.900464)	243.502 *	<0.001 *
	Mean ± SD	143.756068 ^b^ ± 0.314201	146.640054 ^a^ ± 0.248154	146.534000 ^a^ ± 0.297665	146.349573 ^a^ ± 0.253850		
**Lead Angle (°)**	(Min.–Max.)	(11.004825–11.988915)	(11.034662–11.809882)	(11.090000–11.910000)	(10.076297–11.677352)	7.004 *	0.001 *
	Mean ± SD	11.545669 ^a^ ± 0.363683	11.417504 ^a^ ± 0.213034	11.484000 ^a^ ± 0.322359	10.866393 ^b^ ± 0.525236		
**Apical phase angle (°)**	(Min.–Max.)	(56.001507–56.663252)	(56.279845–56.967497)	(56.040000–56.840000)	(56.444012–57.737338)	17.062 *	<0.001 *
	Mean ± SD	56.324464 ^a^ ± 0.227137	56.604501 ^a^ ± 0.219290	56.299000 ^a^ ± 0.247227	57.242810 ^b^ ± 0.539697		

F: F for ANOVA test, pairwise comparison between each two groups was done using Post Hoc Tukey’s test. Means with common letters are not statistically different. * Statistically significant at *p* ≤ 0.05.

**Table 2:** Comparison between the measurements of (Group II) miniscrews calculated by the four software programs.

Group II (HUBIT^®^)		AutoCAD^®^ (*n* = 10)	Image-Pro^®^ (*n* = 10)	MicroDicom (*n* = 10)	Photoshop^®^ (*n* = 10)	F	*p*
**Pitch depth (µm)**	(Min.–Max.)	(254.641–266.926)	(253.790–278.961)	(250.000–270.000)	(260.000–276.843)	3.232 *	0.034 *
	Mean ± SD	261.554 ^a,b^ ± 4.667	260.174 ^a,b^ ± 7.682	256.000 ^b^ ± 8.433	265.455 ^a^ ± 6.043		
**Pitch Width (µm)**	(Min.–Max.)	(820.172–829.820)	(821.603–829.748)	(800.000–820.000)	(814.414–828.829)	22.781 *	<0.001 *
	Mean ± SD	825.958 ^a^ ± 2.722	825.211 ^a^ ± 3.286	812.000 ^b^ ± 6.325	821.334 ^a^ ± 3.741		
**TSF (%)**	(Min.–Max.)	(308.119–323.200)	(306.680–336.378)	(304.878–337.500)	(316.420–339.929)	1.796	0.165
	Mean ± SD	316.666 ± 5.486	315.277 ± 9.014	315.322 ± 11.776	323.223 ± 8.350		
**Flank (µm)**	(Min.–Max.)	(441.528–449.003)	(440.684–449.665)	(440.000–440.000)	(426.635–448.482)	7.811 *	<0.001 *
	Mean ± SD	444.774 ^a,b^ ± 2.746	445.812 ^a^ ± 2.812	440.000 ^b,c^ ± 0.0	436.795 ^c^ ± 8.684		
**Thread Angle (°)**	(Min.–Max.)	(139.014876–139.967791)	(139.195189–139.974292)	(139.170000–139.910000)	(139.030937–139.882863)	1.325	0.281
	Mean ± SD	139.486912 ± 0.314300	139.706386 ± 0.318692	139.518000 ± 0.242065	139.474720 ± 0.306634		
**Lead Angle (°)**	(Min.–Max.)	(8.16005–8.83915)	(8.034495–8.67321398)	(8.020000–8.870000)	(8.02952–8.702224)	1.98	0.134
	Mean ± SD	8.506143 ± 0.285453	8.350101 ± 0.256194	8.5710 ± 0.258175	8.3332 ± 0.249709		
**Apical phase angle (°)**	(Min.–Max.)	(48.085910–48.986046)	(49.165955–50.617764)	(48.050000–48.910000)	(48.142537–48.836248)	42.213 *	<0.001 *
	Mean ± SD	48.542387 ^b^ ± 0.314351	49.923551 ^a^ ± 0.456562	48.416000 ^b^ ± 0.305294	48.508300 ^b^ ± 0.299687		

F: F for ANOVA test, pairwise comparison between each two groups was done using Post Hoc Tukey’s test. Means with common letters are not statistically different. * Statistically significant at *p* ≤ 0.05.

**Table 3:** Comparison between the measurements of (Group III) miniscrews calculated by the four software programs.

Group III (AbsoAnchor^®^)		AutoCAD^®^ (*n* = 10)	Image-Pro^®^ (*n* = 10)	MicroDicom (*n* = 10)	Photoshop^®^ (*n* = 10)	F	*p*
**Pitch depth (µm)**	(Min.–Max.)	(320.137–339.861)	(321.724–338.731)	(320.000–340.000)	(326.788–336.369)	0.800	0.502
	Mean ± SD	333.088 ± 5.978	329.020 ± 6.549	331.000 ± 7.379	330.441 ± 3.062		
**Pitch Width (µm)**	(Min.–Max.)	(710.163–719.808)	713.679 (711.439–716.188)	710.000 (710.000–720.000)	712.778 (710.930–716.477)	0.921	0.440
	Mean ± SD	714.125 ± 3.657	713.704 ± 1.696	712.000 ± 4.216	713.068 ± 1.760		
**TSF (%)**	(Min.–Max.)	(449.686–478.568)	460.409 (451.158–475.950)	464.789 (450.704–478.873)	462.523 (458.586–472.805)	0.691	0.564
	Mean ± SD	466.450 ± 9.361	461.002 ± 9.076	464.906 ± 10.929	463.412 ± 4.712		
**Flank (µm)**	(Min.–Max.)	(390.372–399.690)	375.690 (364.697–387.772)	380.000 (370.000–380.000)	379.237 (371.401–385.901)	27.689 *	<0.001 *
	Mean ± SD	394.003 ^a^ ± 2.992	376.818 ^b^ ± 7.185	376.000 ^b^ ± 5.164	378.165 ^b^ ± 4.288		
**Thread Angle (°)**	(Min.–Max.)	(119.074873–119.855696)	118.561634 (118.050841–118.979835)	118.670000 (118.150000–119.160000)	119.370423 (119.074193–119.979510)	31.057 *	<0.001 *
	Mean ± SD	119.561819 ^a^ ± 0.246613	118.553697 ^b^ ± 0.342074	118.623000 ^b^ ± 0.289177	119.387933 ^a^ ± 0.288939		
**Lead Angle (°)**	(Min.–Max.)	(8.079740–8.985370)	8.4042 (8.074600–8.860830)	8.3000 (8.010000–8.940000)	8.6802 (8.123840–8.921780)	1.59	0.209
	Mean ± SD	8.424602 ± 0.325302	8.444984 ± 0.2245130	8.357000 ± 0.309912	8.623575 ± 0.271516		
**Apical phase angle (°)**	(Min.–Max.)	(29.023804–29.686788)	(29.010168–29.888061)	(28.320000–29.790000)	(28.416172–29.913646)	2.716	0.059
	Mean ± SD	29.396135 ± 0.218475	29.340296 ± 0.319218	28.936000 ± 0.483878	29.198377 ± 0.487591		

F: F for ANOVA test, pairwise comparison between each two groups was done using Post Hoc Tukey’s test. Means with common letters are not statistically different. * Statistically significant at *p* ≤ 0.05.

**Table 4:** Comparison between the measurements of (Group IV) miniscrews calculated by the four software programs.

Group IV(Creative)		AutoCAD^®^ (*n* = 10)	Image-Pro^®^ (*n* = 10)	MicroDicom (*n* = 10)	Photoshop^®^ (*n* = 10)	F	*p*
**Pitch depth (µm)**	(Min.–Max.)	(213.274–225.609)	(212.164–227.032)	(210.000–220.000)	(221.064–227.948)	5.04 *	0.005 *
	Mean ± SD	220.975 ^a,b^ ± 3.537	219.180 ^a,b^ ± 5.464	217.000 ^a^ ± 4.830	224.155 ^b^ ± 2.636		
**Pitch Width (µm)**	(Min.–Max.)	(701.425–708.863)	(703.833–709.227)	(700.000–700.000)	(691.436–708.889)	8.32 *	<0.001 *
	Mean ± SD	704.370 ^a,b^ ± 2.567	706.851 ^a^ ± 2.331	700.000 ^c^ ± 0.0	700.701 ^b,c^ ± 5.458		
**TSF (%)**	(Min.–Max.)	(302.376–320.574)	(299.499–321.960)	(300.000–314.286)	(311.845–328.369)	5.23 *	0.004 *
	Mean ± SD	313.723 ^a^ ± 5.026	310.087 ^b^ ± 7.953	310.000 ^b^ ± 6.901	319.933 ^a,b^ ± 5.505		
**Flank (µm**)	(Min.–Max.)	(230.557–239.658)	(230.214–238.837)	(220.000–230.000)	(231.774–237.836)	13.252 *	<0.001 *
	Mean ± SD	236.215 ^a^ ± 3.339	234.525 ^a^ ± 2.940	228.000 ^b^ ± 4.216	235.532 ^a^ ± 2.340		
**Thread Angle (°)**	(Min.–Max.)	(124.046470–124.046470)	(124.024450–124.893903)	(124.080000–124.970000)	(124.010217–124.924029)	0.490	0.691
	Mean ± SD	124.455527 ± 0.327442	124.355223 ± 0.288327	124.522000 ± 0.329032	124.421630 ± 0.308572		
**Lead Angle (°)**	(Min.–Max.)	(7.103703–7.993718)	(7.004433–7.820794)	(7.040000–7.920000)	(7.070345–7.969916)	0.387	0.763
	Mean ± SD	7.462081 ± 0.290753	7.450476 ± 0.326300	7.586000 ± 0.281749	7.512476 ± 0.350058		
**Apical phase angle (°)**	(Min.–Max.)	(28.250495–28.936429)	(28.250495–28.936429)	(28.050000–28.900000)	(28.156191–28.915424)	0.8310	0.486
	Mean ± SD	28.642882 ± 0.253064	28.642882 ± 0.253064	28.537000 ± 0.289676	28.493861 ± 0.252445		

F: F for ANOVA test, pairwise comparison between each two groups was done using Post Hoc Tukey’s test. Means with common letters are not statistically different. * Statistically significant at *p* ≤ 0.05.

**Table 5:** Intra-assessor reliability of the geometric features’ measurements calculated by AutoCAD^®^ software program for each miniscrew type.

AutoCAD^®^	Variable	ICC	95% CI		Absolute Difference		t	*p*
			Lower	Upper	Mean	SD		
**Group I** (Tomas^®^)	Pitch depth (micron)	0.922	0.719	0.980	0.78	4.21	0.588	0.571
	Pitch width (micron)	0.997	0.987	0.999	0.30	3.06	0.307	0.766
	TSF	0.960	0.847	0.990	0.001	0.005	0.537	0.604
	Flank (micron)	0.659	0.095	0.903	2.42	3.76	2.033	0.073
	Thread angle	−0.408	−0.811	0.257	0.13	0.55	0.766	0.463
	Lead angle	0.420	−0.243	0.816	0.12	0.37	1.035	0.328
	Insertion angle	0.388	−0.280	0.802	0.01	0.28	0.125	0.903
**Group II** (HUBIT^®^)	Pitch depth (micron)	0.412	−0.253	0.813	0.74	5.26	0.445	0.667
	Pitch width (micron)	0.068	−0.557	0.644	0.26	3.61	0.228	0.825
	TSF	0.381	−0.287	0.800	0.001	0.007	0.471	0.649
	Flank (micron)	0.083	−0.546	0.653	0.41	3.59	0.360	0.727
	Thread angle	0.208	−0.451	0.720	0.17	0.36	1.554	0.155
	Lead angle	−0.523	−0.856	0.116	0.17	0.49	1.102	0.299
	Insertion angle	0.067	−0.558	0.643	0.05	0.43	0.365	0.723
**Group III** (AbsoAnchor^®^)	Pitch depth (micron)	0.617	0.024	0.889	0.11	4.67	0.076	0.941
	Pitch width (micron)	−0.431	−0.820	0.231	2.50	5.72	1.385	0.199
	TSF	0.340	−0.329	0.782	0.002	0.009	0.601	0.563
	Flank (micron)	0.163	−0.486	0.697	0.48	4.03	0.373	0.717
	Thread angle	0.283	−0.385	0.756	0.01	0.36	0.115	0.911
	Lead angle	0.118	−0.521	0.672	0.02	0.35	0.200	0.846
	Insertion angle	0.350	−0.320	0.786	0.05	0.33	0.487	0.638
**Group IV** (Creative)	Pitch depth (micron)	0.283	−0.385	0.756	0.86	5.46	0.499	0.630
	Pitch width (micron)	0.603	0.002	0.884	0.20	2.46	0.262	0.799
	TSF	0.401	−0.266	0.808	0.001	0.007	0.495	0.632
	Flank (micron)	0.188	−0.467	0.710	1.45	4.38	1.047	0.322
	Thread angle	−0.203	−0.718	0.454	0.09	0.46	0.649	0.533
	Lead angle	0.018	−0.591	0.613	0.06	0.45	0.456	0.659
	Insertion angle	0.380	−0.288	0.799	0.04	0.24	0.470	0.649

t: Paired *t*-test for comparing between the first and second measurements. * Statistically significant at *p* ≤ 0.05.

**Table 6:** Intra-assessor reliability of the geometric features’ measurements calculated by Image-Pro^®^ software program for each miniscrew type.

Image-Pro^®^	Variable	ICC	95% CI		Absolute Difference		t	*p*
			Lower	Upper	Mean	SD		
**Group I** (Tomas^®^)	Pitch depth (micron)	0.946	0.799	0.986	0.49	5.13	0.301	0.770
	Pitch width (micron)	0.995	0.980	0.999	0.26	3.17	0.256	0.804
	TSF	0.968	0.876	0.992	0.001	0.005	0.330	0.749
	Flank (micron)	0.632	0.049	0.894	0.63	2.02	0.978	0.354
	Thread angle	0.226	−0.436	0.729	0.26	0.37	2.255	0.051
	Lead angle	0.494	−0.154	0.845	0.16	0.23	2.134	0.062
	Insertion angle	0.010	−0.596	0.608	0.12	0.36	1.052	0.320
**Group II** (HUBIT^®^)	Pitch depth (micron)	0.896	0.639	0.973	3.67	3.65	3.181 *	0.011 *
	Pitch width (micron)	0.135	−0.509	0.682	1.15	3.92	0.928	0.378
	TSF	0.823	0.437	0.953	0.004	0.006	2.205	0.055
	Flank (micron)	0.561	−0.063	0.869	0.25	2.51	0.311	0.763
	Thread angle	−0.424	−0.818	0.239	0.23	0.53	1.379	0.201
	Lead angle	−0.072	−0.646	0.554	0.11	0.34	0.982	0.352
	Insertion angle	0.814	0.416	0.950	0.06	0.28	0.709	0.496
**Group III** (AbsoAnchor^®^)	Pitch depth (micron)	0.768	0.309	0.937	0.16	4.09	0.123	0.905
	Pitch width (micron)	0.332	−0.337	0.779	2.79	1.53	5.756 *	<0.001 *
	TSF	0.799	0.378	0.946	0.002	0.005	0.951	0.367
	Flank (micron)	0.767	0.306	0.937	2.41	4.44	1.715	0.120
	Thread angle	0.248	−0.416	0.740	0.09	0.39	0.687	0.510
	Lead angle	−0.577	−0.875	0.038	0.06	0.41	0.479	0.643
	Insertion angle	−0.018	−0.614	0.590	0.23	0.40	1.825	0.101
**Group IV** (Creative)	Pitch depth (micron)	0.872	0.568	0.967	0.70	3.14	0.706	0.498
	Pitch width (micron)	−0.244	−0.738	0.420	1.10	3.45	1.011	0.338
	TSF	0.846	0.498	0.960	0.001	0.005	0.934	0.375
	Flank (micron)	0.479	−0.173	0.839	0.94	3.15	0.943	0.370
	Thread angle	0.259	−0.407	0.745	0.10	0.36	0.893	0.395
	Lead angle	−0.195	−0.714	0.461	0.05	0.45	0.371	0.719
	Insertion angle	0.488	−0.161	0.843	0.24	0.27	2.883 *	0.018 *

t: Paired *t*-test for comparing between the first and second measurements. * Statistically significant at *p* ≤ 0.05.

**Table 7:** Intra-assessor reliability of the geometric features’ measurements calculated by MicroDicom software program for each miniscrew type.

MicroDicom	Variable	ICC	95% CI		Absolute Difference		t	*p*
			Lower	Upper	Mean	SD		
**Group I** (Tomas^®^)	Pitch depth (micron)	1.000	1.000	1.000	0.0	0.0	–	–
	Pitch width (micron)	1.000	1.000	1.000	0.0	0.0	–	–
	TSF	1.000	1.000	1.000	0.0	0.0	–	–
	Flank (micron)	1.000	1.000	1.000	0.0	0.0	–	–
	Thread angle	−0.423	−0.817	0.241	0.08	0.46	0.564	0.587
	Lead angle	0.265	−0.401	0.748	0.21	0.34	1.906	0.089
	Insertion angle	0.386	−0.282	0.802	0.13	0.26	1.591	0.146
**Group II** (HUBIT^®^)	Pitch depth (micron)	1.000	1.000	1.000	0.0	0.0	–	–
	Pitch width (micron)	1.000	1.000	1.000	0.0	0.0	–	–
	TSF	1.000	1.000	1.000	0.0	0.0	–	–
	Flank (micron)	1.000	1.000	1.000	0.0	0.0	–	–
	Thread angle	0.280	−0.387	0.755	0.15	0.28	1.651	0.133
	Lead angle	−0.432	−0.821	0.229	0.03	0.34	0.296	0.774
	Insertion angle	−0.297	−0.763	0.371	0.12	0.43	0.890	0.397
**Group III** (AbsoAnchor^®^)	Pitch depth (micron)	1.000	1.000	1.000	0.0	0.0	–	–
	Pitch width (micron)	1.000	1.000	1.000	0.0	0.0	–	–
	TSF	1.000	1.000	1.000	0.0	0.0	–	–
	Flank (micron)	1.000	1.000	1.000	0.0	0.0	–	–
	Thread angle	0.668	0.111	0.906	0.12	0.33	1.113	0.295
	Lead angle	0.557	−0.068	0.868	0.37	0.27	4.375 *	0.002 *
	Insertion angle	0.723	0.214	0.923	0.03	0.34	0.279	0.787
**Group IV** (Creative)	Pitch depth (micron)	1.000	1.000	1.000	0.0	0.0	–	–
	Pitch width (micron)	0.0	−0.602	0.602	5.0	5.27	3.0 *	0.015 *
	TSF	0.943	0.787	0.985	0.002	0.002	2.999 *	0.015 *
	Flank (micron)	1.000	1.000	1.000	0.0	0.0	–	–
	Thread angle	−0.178	−0.704	0.475	0.11	0.47	0.711	0.495
	Lead angle	−0.575	−0.874	0.041	0.15	0.48	0.959	0.362
	Insertion angle	−0.265	−0.748	0.401	0.10	0.39	0.833	0.426

t: Paired *t*-test for comparing between the first and second measurements. * Statistically significant at *p* ≤ 0.05.

**Table 8:** Intra-assessor reliability of the geometric features’ measurements calculated by Photoshop^®^ software program for each miniscrew type.

Photoshop^®^	Variable	ICC	95% CI		Absolute Difference		t	*p*
			Lower	Upper	Mean	SD		
**Group I** (Tomas^®^)	Pitch depth (micron)	0.911	0.685	0.977	1.50	3.86	1.230	0.250
	Pitch width (micron)	0.997	0.989	0.999	2.31	2.53	2.897 *	0.018 *
	TSF	0.949	0.809	0.987	0.002	0.004	1.660	0.131
	Flank (micron)	0.855	0.521	0.962	0.37	1.38	0.843	0.421
	Thread angle	0.346	−0.323	0.785	0.21	0.31	2.201	0.055
	Lead angle	0.732	0.232	0.926	0.08	0.47	0.565	0.586
	Insertion angle	0.762	0.295	0.935	0.01	0.35	0.059	0.955
**Group II** (HUBIT^®^)	Pitch depth (micron)	0.826	0.446	0.954	2.18	2.88	2.401 *	0.040 *
	Pitch width (micron)	0.308	−0.361	0.768	2.43	4.95	1.553	0.155
	TSF	0.856	0.525	0.962	0.002	0.004	1.408	0.193
	Flank (micron)	0.930	0.746	0.982	2.22	3.16	2.218	0.054
	Thread angle	0.667	0.108	0.905	0.05	0.24	0.631	0.544
	Lead angle	0.882	0.597	0.969	0.04	0.12	0.937	0.373
	Insertion angle	0.403	−0.263	0.809	0.12	0.26	1.451	0.181
**Group III** (AbsoAnchor^®^)	Pitch depth (micron)	0.415	−0.249	0.814	0.47	3.07	0.489	0.637
	Pitch width (micron)	−0.278	−0.754	0.389	2.83	2.76	3.237 *	0.010 *
	TSF	0.327	−0.343	0.776	0.003	0.005	1.583	0.148
	Flank (micron)	0.689	0.148	0.912	1.85	3.69	1.584	0.148
	Thread angle	−0.005	−0.605	0.599	0.0	0.3	0.192	0.852
	Lead angle	0.124	−0.517	0.675	0.2	0.4	1.648	0.134
	Insertion angle	0.551	−0.077	0.866	0.2	0.5	1.138	0.284
**Group IV** (Creative)	Pitch depth (micron)	0.757	0.285	0.934	1.07	1.83	1.843	0.098
	Pitch width (micron)	0.769	0.311	0.937	1.05	3.40	0.982	0.352
	TSF	0.802	0.386	0.947	0.001	0.003	1.028	0.331
	Flank (micron)	0.114	−0.524	0.670	0.71	3.72	0.606	0.560
	Thread angle	0.138	−0.506	0.683	0.03	0.37	0.258	0.803
	Lead angle	−0.299	−0.763	0.370	0.04	0.53	0.212	0.837
	Insertion angle	0.093	−0.539	0.658	0.02	0.38	0.159	0.877

t: Paired *t*-test for comparing between the first and second measurements. * Statistically significant at *p* ≤ 0.05.

**Table 9:** Intra-assessor reliability of the geometric features’ measurements combined for the four types of miniscrews by each software program.

Software	Variable	ICC	95% CI		t	*p*
			Lower	Upper		
**AutoCAD^®^**	Pitch depth (micron)	0.995	0.990	0.997	0.756	0.454
	Pitch width (micron)	0.999	0.998	0.999	1.111	0.274
	TSF	0.996	0.993	0.998	0.267	0.791
	Flank (micron)	0.999	0.998	0.999	0.350	0.729
	Thread angle	0.999	0.998	1.000	0.142	0.888
	Lead angle	0.963	0.932	0.980	0.352	0.727
	Apical face angle	1.000	0.999	1.000	0.133	0.895
**Image-Pro^®^**	Pitch depth (micron)	0.995	0.991	0.998	1.345	0.186
	Pitch width (micron)	0.999	0.999	1.000	2.648 *	0.012 *
	TSF	0.997	0.995	0.999	0.419	0.678
	Flank (micron)	0.999	0.999	1.000	1.847	0.072
	Thread angle	0.999	0.999	1.000	1.772	0.084
	Lead angle	0.973	−0.243	0.816	0.634	0.530
	Apical face angle	1.000	0.999	1.000	0.830	0.411
**MicroDicom**	Pitch depth (micron)	1.000	1.000	1.000	–	–
	Pitch width (micron)	0.999	0.998	0.999	2.360 *	0.023 *
	TSF	1.000	1.000	1.000	2.360 *	0.023 *
	Flank (micron)	1.000	1.000	1.000	–	–
	Thread angle	0.999	0.999	1.000	0.024	0.981
	Lead angle	0.967	0.938	0.982	2.874 *	0.007 *
	Apical face angle	1.000	0.999	1.000	1.461	0.152
**Photoshop^®^**	Pitch depth (micron)	0.997	0.995	0.999	0.628	0.534
	Pitch width (micron)	0.999	0.998	1.000	3.937 *	0.001 *
	TSF	0.998	0.997	0.999	0.739	0.465
	Flank (micron)	0.999	0.999	1.000	2.614 *	0.013 *
	Thread angle	1.000	0.999	1.000	1.569	0.125
	Lead angle	0.957	0.920	0.977	1.404	0.168
	Apical face angle	1.000	0.999	1.000	0.178	0.860

t: Paired ***t***-test for comparing between the first and second measurements. * Statistically significant at ***p*** ≤ 0.05.

## Data Availability

All data are available upon request by contacting the Authors.

## References

[1] Kamburoglu K., Barenboim S.F., Aritürk T., Kaffe I. (2008). Quantitative measurements obtained by micro-computed tomography and confocal laser scanning microscopy. Dentomaxillofac. Radiol..

[2] Frederick K.K., Karla Z., Thiago d.A.P.N.C., Murilo N.d.O., Caio C.D.R., Flávio D.d.N. (2017). Comparative analysis of Optical Microscopy, Scanning Electron Microscopy, and Micro-Computed Tomography on measurements. BJOS.

[3] Silva A.A.L.S., Franco A., Fernandes Â., Costa C., Barbosa J.S., Westphalen F.H. (2017). Accuracy of linear measurements performed with two imaging software in cone-beam computed tomography scans of dry human mandibles. An. Acad. Bras. Cienc..

[4] El H., Palomo J.M. (2010). Measuring the airway in 3 dimensions: A reliability and accuracy study. Am. J. Orthod. Dentofac. Orthop..

[5] Weissheimer A., Menezes L.M., Sameshima G.T., Enciso R., Pham J., Grauer D. (2012). Imaging software accuracy for 3-dimensional analysis of the upper airway. Am. J. Orthod. Dentofac. Orthop..

[6] Mantovani E., Castroflorio E., Rossini G., Garino F., Cugliari G., Deregibus A., Castroflorio T. (2018). Scanning electron microscopy evaluation of aligner fit on teeth. Angle Orthod..

[7] Döler W., Steinhöfel N., Jäger A. (1991). Digital image processing techniques for cephalometric analysis. Comput. Biol. Med..

[8] Farooq M.U., Khan M.A., Imran S., Sameera A., Qureshi A., Ahmed S.A., Kumar S., Rahman M.A. (2016). Assessing the reliability of digitalized cephalometric analysis in comparison with manual cephalometric analysis. J. Clin. Diagn. Res..

[9] Jin P., Li X. (2015). Correction of image drift and distortion in a scanning electron microscopy. J. Microsc..

[10] Quieregatto P.R., Hochman B., Furtado F., Machado A.F., Sabino Neto M., Ferreira L.M. (2014). Image analysis software versus direct anthropometry for breast measurements. Acta Cir. Bras..

[11] Chen H., van Eijnatten M., Wolff J., de Lange J., van der Stelt P.F., Lobbezoo F., Aarab G. (2017). Reliability and accuracy of three imaging software packages used for 3D analysis of the upper airway on cone beam computed tomography images. Dentomaxillofac. Radiol..

[12] Pachêco-Pereira C., Alsufyani N., Major M., Palomino-Gómez S., Pereira J.R., Flores-Mir C. (2017). Correlation and reliability of cone-beam computed tomography nasopharyngeal volumetric and area measurements as determined by commercial software against nasopharyngoscopy-supported diagnosis of adenoid hypertrophy. Am. J. Orthod. Dentofac. Orthop..

[13] Shahidi S., Bahrampour E., Soltanimehr E., Zamani A., Oshagh M., Moattari M., Mehdizadeh A. (2014). The accuracy of a designed software for automated localization of craniofacial landmarks on CBCT images. BMC Med. Imaging.

[14] Störchle P., Müller W., Sengeis M., Ahammer H., Fürhapter-Rieger A., Bachl N., Lackner S., Mörkl S., Holasek S. (2017). Standardized ultrasound measurement of subcutaneous fat patterning: High reliability and accuracy in groups ranging from lean to obese. Ultrasound Med. Biol..

[15] Cotrim-Ferreira A., Cotrim-Ferreira F., Vellini-Ferreira F., Peron D.F., Carvalho C., Torres F.C. (2014). Evaluation of cervico-occlusal dimensions of maxillary and mandibular incisor brackets for lingual orthodontics. J. Contemp. Dent. Pract..

[16] Dolci G.S., Spohr A.M., Zimmer E.R., Marchioro E.M. (2013). Assessment of the dimensions and surface characteristics of orthodontic wires and bracket slots. Dent. Press J. Orthod..

[17] da Cunha A.C., Marquezan M., Lima I., Lopes R.T., Nojima L.I., Sant’Anna E.F. (2015). Influence of bone architecture on the primary stability of different mini-implant designs. Am. J. Orthod. Dentofac. Orthop..

[18] da Cunha A.C., Marquezan M., Nojima L.I., Sant’Anna E.F. (2017). Evaluation of mechanical performance of orthodontic mini-implants with distinct designs. Iran. J. Orthop. Surg..

[19] Radwan E.S., Montasser M.A., Maher A. (2018). Influence of geometric design characteristics on primary stability of orthodontic miniscrews. J. Orofac. Orthop..

[20] Postek M.T. (1994). Critical Issues in Scanning Electron Microscope Metrology. J. Res. Natl. Inst. Stand. Technol..

[21] Postek M.T., Vladár A.E. (2013). Does your SEM really tell the truth?—How would you know? Part 1. Scanning.

[22] Postek M.T., Vladár A.E., Purushotham K.P. (2014). Does your SEM really tell the truth? How would you know? Part 2. Scanning.

[23] Thong J.T., Lee K.W., Wong W.K. (2001). Reduction of charging effects using vector scanning in the scanning electron microscope. Scanning.

[24] de Faria M.G., Haddab Y., Le Gorrec Y., Lutz P. (2015). Influence of mechanical noise inside a scanning electron microscope. Rev. Sci. Instrum..

[25] Lee K.M., Lee J., Chung C.Y., Ahn S., Sung K.H., Kim T.W., Lee H.J., Park M.S. (2012). Pitfalls and important issues in testing reliability using intraclass correlation coefficients in orthopaedic research. Clin. Orthop. Surg..

[26] McGraw K.O., Wong S.P. (1996). Forming inferences about some intraclass correlation coefficients. Psychol. Methods.

[27] Fisher R.A. (1921). On the probable error of a coefficient of correlation deduced from a small sample. Metron.

[28] Koo T.K., Li M.Y. (2017). A Guideline of Selecting and Reporting Intraclass Correlation Coefficients for Reliability Research. J. Chiropr. Med..

[29] Portney L.G., Watkins M.P. (2000). Foundations of Clinical Research: Applications to Practice.

[30] Cole T.J. (2015). Too many digits: The presentation of numerical data. Arch. Dis. Child..

[31] Altman D.G., Bland J.M. (1996). Statistics notes 15. Presentation of numerical data. BMJ.

[32] Lo Giudice A., Quinzi V., Ronsivalle V., Martina S., Bennici O., Isola G. (2020). Description of a Digital Work-Flow for CBCT-Guided Construction of Micro-Implant Supported Maxillary Skeletal Expander. Materials (Basel).

[33] Scribante A., Montasser M.A., Radwan E.S., Bernardinelli L., Alcozer R., Gandini P., Sfondrini M.F. (2018). Reliability of Orthodontic Miniscrews: Bending and Maximum Load of Different Ti-6Al-4V Titanium and Stainless Steel Temporary Anchorage Devices (TADs). Materials (Basel).

[34] Sfondrini M.F., Gandini P., Alcozer R., Vallittu P.K., Scribante A. (2018). Failure load and stress analysis of orthodontic miniscrews with different transmucosal collar diameter. J. Mech. Behav. Biomed. Mater..

